# Management of an Incomplete Pharyngocutaneous Fistula Following Salvage Laryngectomy With Polyethylene Glycol Hydrogel Sealant

**DOI:** 10.7759/cureus.105846

**Published:** 2026-03-25

**Authors:** Lentiona Basiari, Charikleia Koutsikou, Alexandros Georgolios, Georgios V Psychogios

**Affiliations:** 1 Department of Otorhinolaryngology, Head and Neck Surgery, University Hospital of Ioannina, Ioannina, GRC; 2 Department of Otorhinolaryngology, University of Ioannina, Ioannina, GRC; 3 Department of Otolaryngology-Head and Neck Surgery, University of Ioannina, Ioannina, GRC

**Keywords:** endoscopic approach, fistula, laryngectomy complications, pharyngocutaneous fistula, total laryngectomy

## Abstract

Pharyngocutaneous fistula (PCF) is a common complication following total laryngectomy, significantly affecting patients' postoperative courses. Its management is challenging, encompassing a broad spectrum of approaches, ranging from conservative measures to more invasive surgical interventions.

A 75-year-old male status post salvage total laryngectomy with modified radical bilateral neck dissection after failure of definitive radiotherapy for squamous cell carcinoma of the larynx developed an incomplete pharyngocutaneous fistula postoperatively. Despite the conservative care during postoperative care, the incomplete fistula didn’t resolve after a month. Through rigid pharyngoesophagoscopy, polyethylene glycol hydrogel was injected into the orifice of the fistula. A follow-up barium swallow study four weeks post-intervention revealed complete resolution of the fistula.

Polyethylene glycol hydrogel sealant, primarily approved for neurosurgical and spinal applications, can be considered off-label for the management of pharyngocutaneous fistulas.

## Introduction

Laryngeal cancer is one of the main causes of morbidity and mortality worldwide. Although in the last decades treatment of laryngeal cancer, especially early laryngeal cancer, has shifted towards organ preservation therapies, total laryngectomy remains an effective treatment for advanced laryngeal cancer and as a salvage procedure after failure of organ preservation surgeries (transoral laser resection, partial laryngectomy) or definitive radiotherapy with or without chemotherapy.

One of the most challenging complications of postlaryngectomy is the development of a pharyngocutaneous fistula (PCF). A PCF results from a dehiscence of the reconstructed neopharynx, leading to the drainage of saliva to the skin and creation of a fistula. The incidence of pharyngocutaneous fistula after total laryngectomy is reported between 9 and 25% after primary surgery and 30 and 70% after salvage surgery [1].

The most important risk factors for the development of a PC fistula include a history of radiation therapy, chemotherapy, and diabetes. Other contributing factors may include patient medical comorbidities, nutritional deficiency, and significant weight loss before surgery, advanced tumor stage, previous tracheostomy, positive resection margins, and the surgeon's experience [2].

Various methods have been proposed for the treatment of this serious complication, ranging from conservative to more complex surgical interventions. Conservative therapies include compressive dressings, intravenous antibiotics, avoidance of oral feeding, negative pressure (vacuum-assisted), gamma, and botulinum toxin injections [3]. Surgical options include pedicled or free flaps to reinforce pharyngeal closure. A recently reported innovative method in the literature includes endoscopic treatment of pharyngocutaneous fistula with the use of plasma-rich compounds [4].

An incomplete pharyngocutaneous fistula refers to a tract that forms between the neopharynx and the skin but does not fully communicate with the external surface of the neck. The incomplete fistula may end blindly in the soft tissues or present as a localized collection near the surgical site. In the present case report, we set out to present our experience with the successful management of an incomplete pharyngocutaneous fistula treated through an endoscopic approach with the use of polyethylene glycol hydrogel sealant.

## Case presentation

We present the case of a 75-year-old male with a history of residual squamous cell carcinoma of the larynx (rcT3cN0cM0) after definitive radiation therapy (66 Gy). The initial diagnosis of squamous cell carcinoma of the right true vocal cord was set in May 2024. The patient completed definitive radiation therapy in August 2024. In 10/2024, the patient underwent microlaryngoscopy under general anesthesia, and new biopsies revealed moderately differentiated squamous cell carcinoma. He was subsequently admitted to our facility for a scheduled salvage total laryngectomy with modified bilateral neck dissection. After tumor resection, frozen section analysis was used intraoperatively to assess the margins of resection, which turned out negative for malignancy. The neopharynx was reconstructed with a T-shaped closure. The closure involved three layers: mucosa, constrictor pharyngeal muscles, and staph muscles, and was reinforced with a thrombin-coated collagen sealant patch and Vicryl 3.0 sutures.

**Figure 1:**
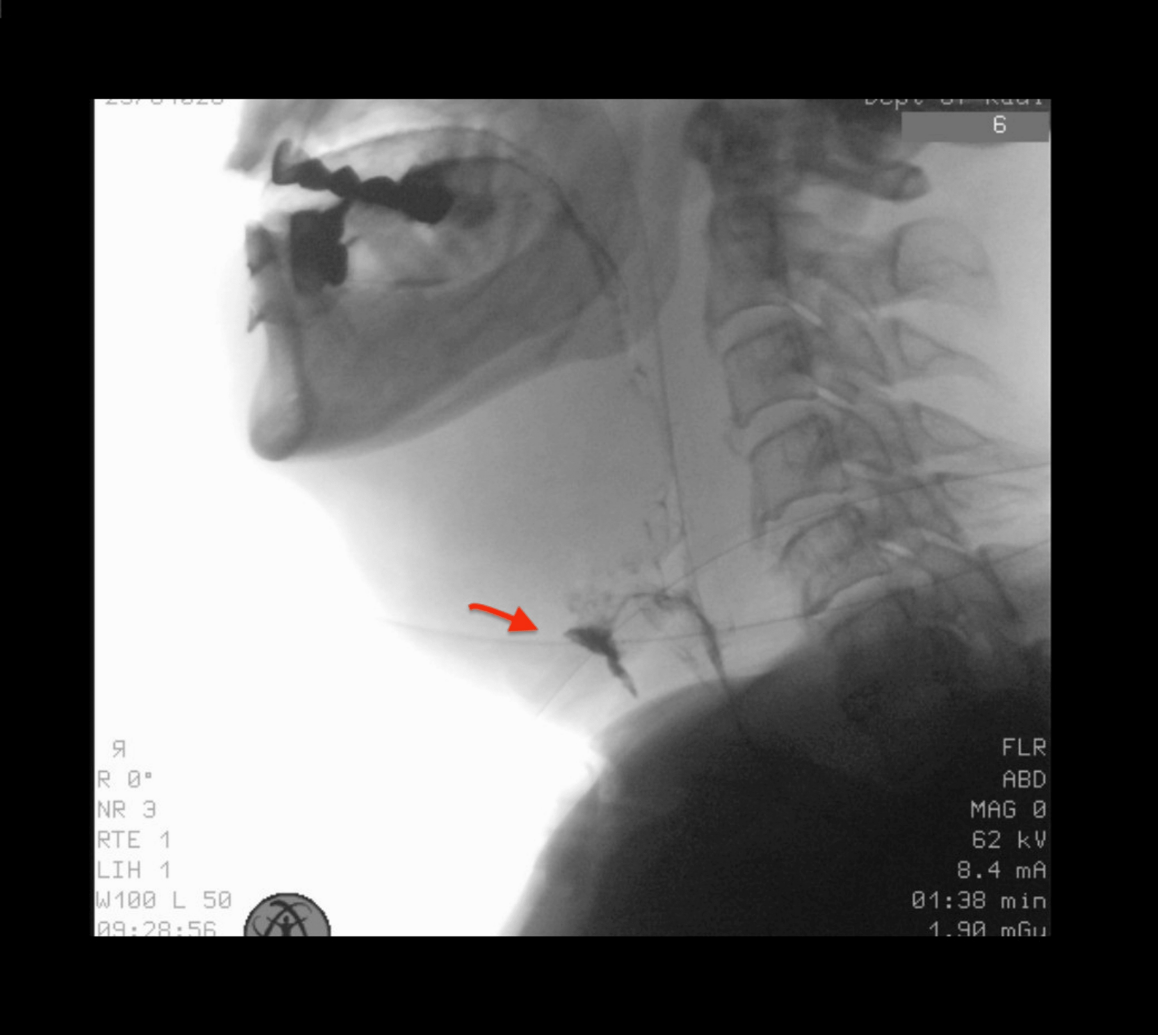
Presence of an incomplete pharyngocutaneous fistula on the barium swallow test on the 17th postlaryngectomy day (arrow)

Postoperatively, the patient received parenteral feeding followed by gradual enteral feeding through a nasogastric tube. In our department, in patients with a total laryngectomy, we perform a barium swallow usually on the 9th postoperative day before starting oral feeding. The patient was diagnosed with an incomplete pharyngocutaneous fistula on the 17th postlaryngectomy day during a barium swallow study, which in this patient was delayed due to a history of prior radiotherapy (Figure 1).

**Figure 2:**
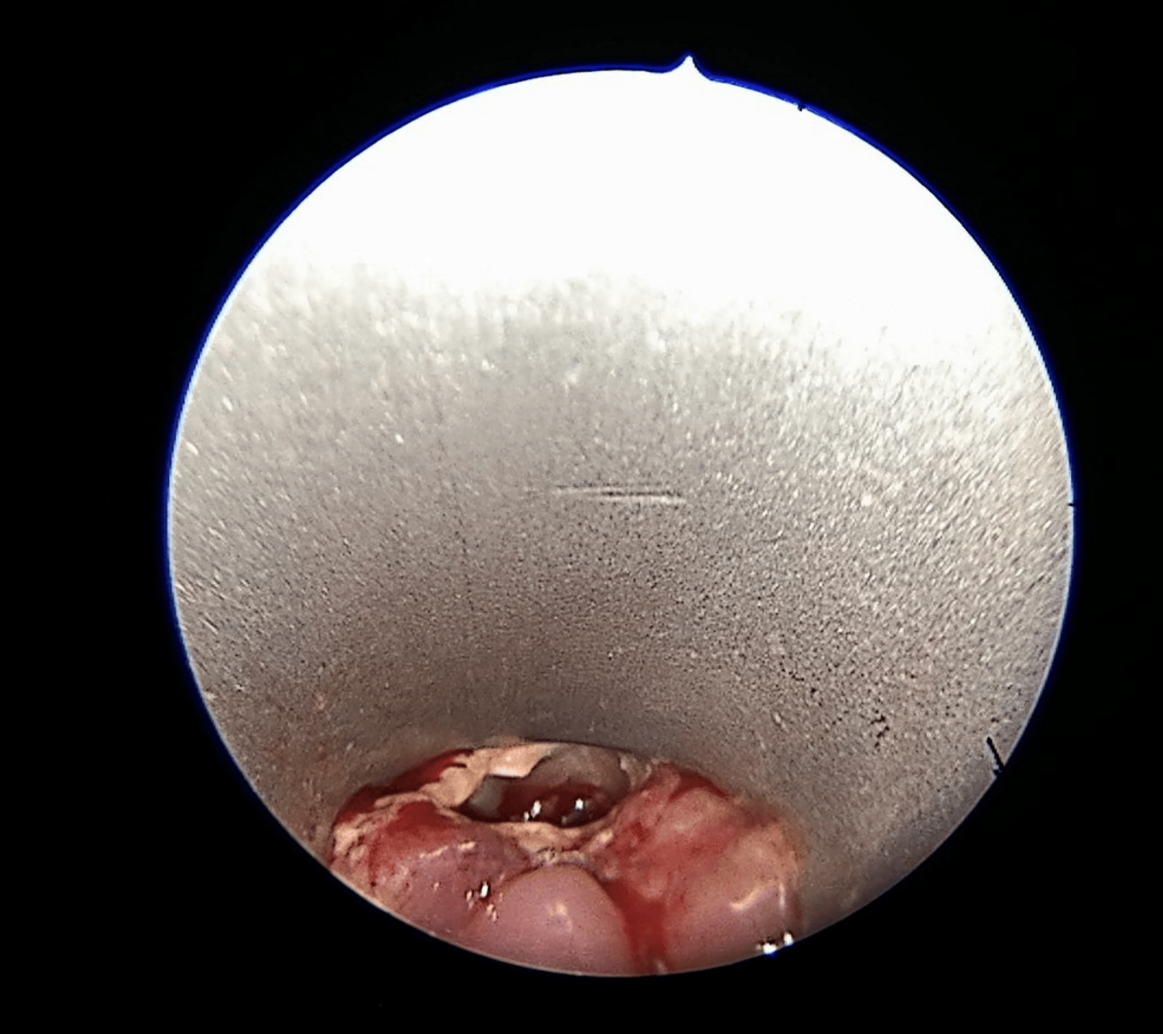
Endoscopic photo of the fistula, localized on the anterior wall of the neopharynx

This was confirmed through endoscopy, which revealed the small internal fistula orifice to the reconstructed neopharynx (Figure 2).

Conservative management included pressure dressings and intravenous broad-spectrum antibiotics. A follow-up swallowing study on the 30th postoperative day showed no changes of incomplete pharyngocutaneous fistula. Due to the failure of the conservative management, the patient was scheduled for placement of a percutaneous endoscopic gastrostomy tube (PEG) and for transoral examination and repair of the fistula. Thus, the patient was taken to the operating room, and an endoscopy was initiated under general anesthesia.

At first, percutaneous endoscopic gastrostomy was performed in collaboration with the gastroenterologist. Through flexible endoscopy, the leakage of the mucosa that caused the fistula was recognized, and subsequently, through a transoral approach using a Kleinsasser laryngoscope combined with a rigid 0-degree endoscope, the orifice of the fistula was localized on the anterior wall of the neopharynx.

**Figure 3:**
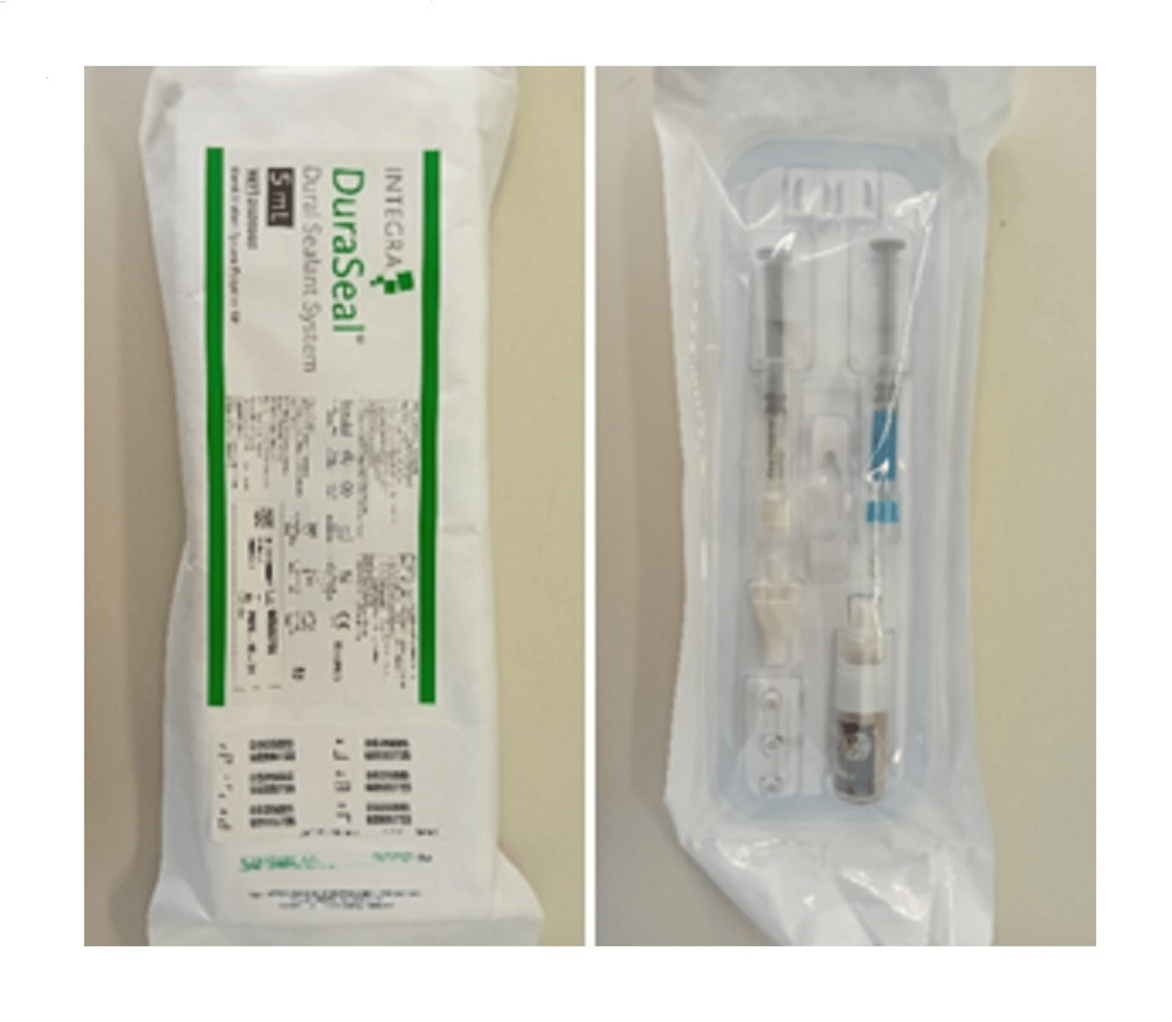
Sealant polyethylene glycol hydrogel set

First, the area was cleaned with a saline solution, the internal aspect of the fistula was debrided, and its edges were freshened. The dehiscence had a diameter of approximately 1 cm, and after meticulous preparation, DuraSeal was injected with a modified intermittent Nelaton catheter. The compound used is called DuraSeal™, a polyethylene glycol hydrogel used during cranial surgery to strengthen dural repairs with watertight closure and support the healing process [5]. The DuraSeal hydrogel can swell up to 50% of its size in any direction (Figure 3).

**Figure 4:**
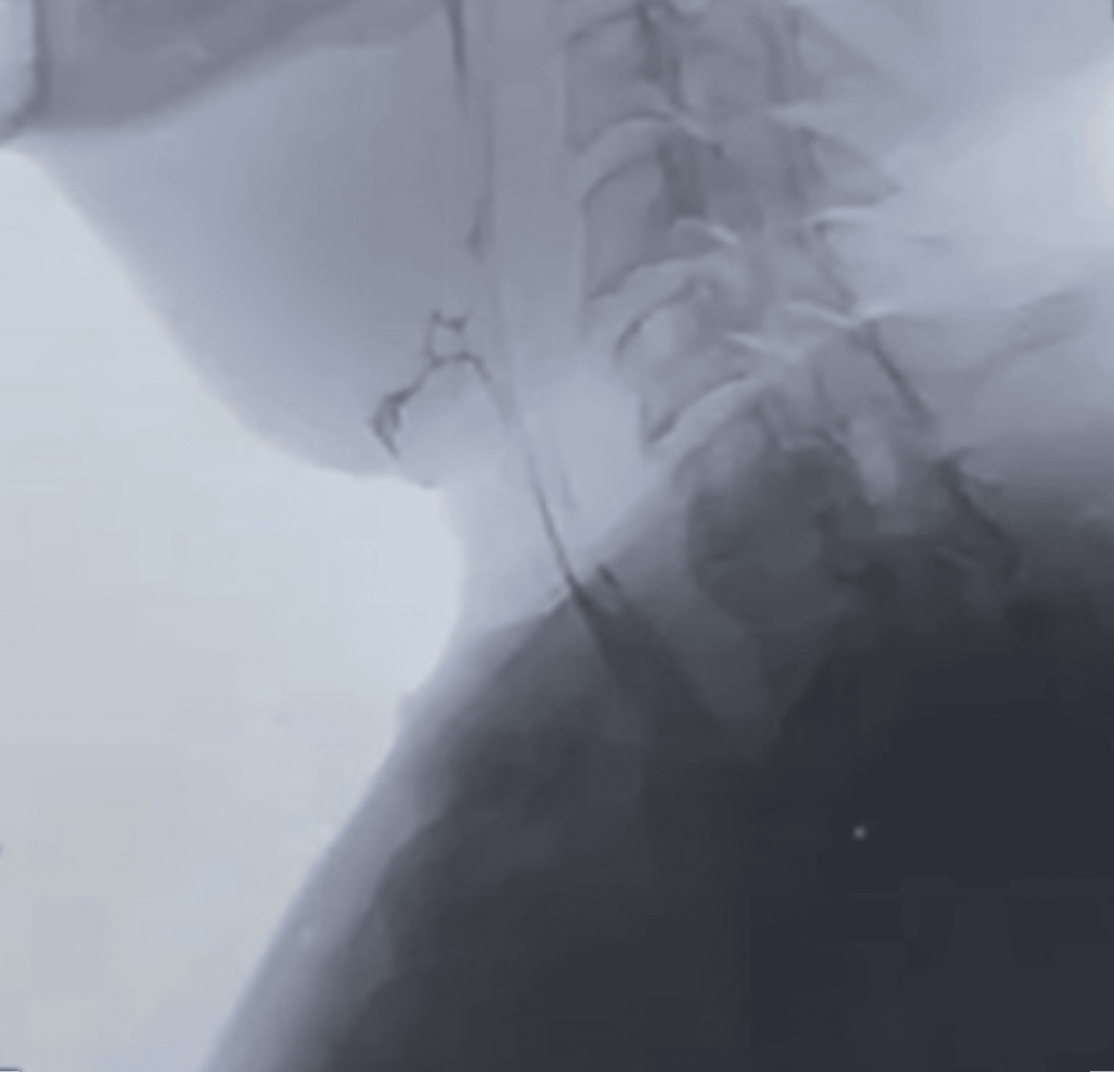
Closure of pharyngocutaneous fistula on barium swallow test on the 25th postendoscopy day with minimal stagnation of barium-diluted food on the reconstructed pharynx

On the 25th postopendoscopy day (55 days after initial salvage laryngectomy) (Figure 4), through a barium swallow study, no contrast extravasation was observed, and the incomplete fistula was significantly reduced to almost complete resolution.

## Discussion

The most common and challenging complication following total laryngectomy is the formation of a pharyngocutaneous fistula. This condition increases morbidity, prolongs hospitalization and recovery time, delays the initiation of adjuvant therapy, and overall, severely impacts the patient’s quality of life. The incidence of pharyngocutaneous fistulas ranges from 3% to 65%, depending on various contributing factors. To better understand these multifactorial influences, they can be categorized into patient-related, disease-related, and treatment-related factors.

Patient-related factors include age >60 years and comorbidities such as heart failure, diabetes mellitus, COPD, and nutritional status (e.g., low albumin levels and low hemoglobin levels). Malnutrition must be identified and corrected during preoperative preparation. Several studies have shown that patients who received arginine-supplemented immunonutrition demonstrated a reduction in pharyngocutaneous fistula and postoperative pneumonia, although no significant effect was observed on wound infections, free flap failure, or bleeding [6].

Another important subject is postoperative feeding and nutrition. While early oral feeding is associated with an increased risk of pharyngocutaneous fistulas, oral hydration may help reduce this risk by promoting mechanical removal of stagnant saliva at the suture site of the reconstructed neopharynx [7].

Early detection of pharyngocutaneous fistulas is essential for effective management. A commonly used method is the blue dye oral test, which is both cost-effective and easily repeatable. In our case, we favored a postoperative barium swallow study, a diagnostic procedure where diluted-barium-infused food is ingested, and the swallowing function is assessed for irregularities or contrast leakage during simultaneous fluoroscopy. The appropriate time for a barium swallow test has not studied enough. Timing may vary from 7-9 days to 12-14 days, depending on the patient's risk factors like previous radiotherapy or chemotherapy and the attending doctor’s preference [8]. In our institution, a test is ordered on the 9th postoperative day after primary laryngectomy and after two weeks after salvage laryngectomy in order to provide appropriate healing of the neopharynx.

Conservative treatment approaches for pharyngocutaneous fistulas include broad-spectrum antibiotics, compressive dressings, and a combination of parenteral nutrition or feeding via nasogastric tube [9]. Additional non-surgical interventions such as hyperbaric oxygen therapy (HBOT), vacuum-assisted closure (VAC), or botulinum toxin therapy have shown some promise in achieving satisfactory closure of post-laryngectomy fistulas.

Botulinum toxin therapy is an established pharmacologic option for reducing salivary secretion, a contributing factor in the formation of pharyngocutaneous fistulas [10]. When administered via periparotid injection, the effect typically begins around 72 hours post-injection and becomes more noticeable between 5 and 7 days [11]. The salivary suppression is reversible within 2 to 4 months and is generally well tolerated with minimal reported side effects.

While traditional reconstruction techniques, such as free or pedicled flaps, remain effective in managing large, complex fistulas, they are often associated with high complication rates, including refistulization, wound dehiscence, and increased morbidity [12]. These approaches are particularly challenging in cases with infected, disrupted tissues and prior radiation exposure. In contrast, smaller fistulas that do not require extensive reconstruction can be managed successfully with less invasive interventions.

Recent studies have highlighted the effectiveness of endoscopic management in treating post-laryngectomy pharyngocutaneous fistulas. A key factor impeding spontaneous closure is the persistent presence of saliva at the orifice of the reconstructed neopharynx, which prevents tissue healing. By sealing the internal orifice of the fistula, salivary flow is excluded, creating favorable conditions for surrounding tissues to fuse and regenerate. One report demonstrated success using autologous fat injection around the hypopharyngeal opening of the PC fistula to compress the orifice and promote wound healing [13]. Plasma rates fibrin glue has also been used successfully for the same purpose by other authors [14].

In our case, we applied endoscopically a polyethylene glycol hydrogel to manage a small, incomplete fistula, following previous conservative management. The fistula was detected early, before extensive epithelialization or scarring had occurred, allowing for precise, minimally invasive intervention. The success of this approach emphasizes the importance of timely diagnosis and careful selection of the sealing agent. While this case demonstrates feasibility and potential effectiveness, further research is necessary to assess the efficacy and safety of various materials used for this intervention. DuraSeal™ is a very safe, well-established material that has received FDA approval as an adjunct to suture dural repair during cranial procedures. There is no current FDA-approved indication for the use of DuraSeal™ in the management of PC fistulas. However, there are anecdotal reports and off-label applications in otolaryngology where similar sealing agents, such as plasma-rich fibrin (PRF), have been employed to promote good healing and fistula closure. The current literature, as well as the risks/benefits/alternatives of the intervention, was extensively discussed with the patient prior to proceeding, and informed consent was obtained. To our knowledge, this is the first report of successful treatment of an incomplete pharyngocutaneous fistula using the specific endoscopic technique.

## Conclusions

In cases where conservative management fails, surgical intervention becomes essential for the treatment of pharyngocutaneous fistulas. Although reconstructive procedures using pedicled or free flaps are effective, they are often associated with high complication rates, including re-fistulization and prolonged recovery. As a less invasive alternative, endoscopic techniques utilizing sealing compounds offer a promising direction but remain underexplored. Among them, DuraSeal™ sealant, though originally developed for cranial applications, has demonstrated potential in the successful management of incomplete pharyngocutaneous fistulas. However, its broader clinical application requires further validation through larger case series and prospective studies to assess long-term efficacy and safety.

## References

[1] Sittitrai P, Srivanitchapoom C, Reunmakkaew D (2018). Prevention of pharyngocutaneous fistula in salvage total laryngectomy: role of the pectoralis major flap and peri-operative management. J Laryngol Otol.

[2] Paydarfar JA, Birkmeyer NJ (2006). Complications in head and neck surgery: a meta-analysis of postlaryngectomy pharyngocutaneous fistula. Arch Otolaryngol Head Neck Surg.

[3] Rao KN, Arora RD, Singh A (2022). Pharyngocutaneous fistula following primary total laryngectomy: a meta-analysis. Indian J Surg Oncol.

[4] Başkadem Yilmazer A, Aksungur E, Çelik C (2025). Results of platelet-rich fibrin application in pharyngeal reconstruction after a total laryngectomy. Clin Otolaryngol.

[5] (2026). DuraSeal: Dura Sealant. https://products.integralife.com/duraseal-dural-sealant-system/product/dural-repair-sealants-duraseal-dural-sealant-system-5-ml.

[6] Nesemeier R, Dunlap N, McClave SA, Tennant P (2017). Evidence-based support for nutrition therapy in head and neck cancer. Curr Surg Rep.

[7] Singh R, Karantanis W, Fadhil M (2021). Meta-analysis on the rate of pharyngocutaneous fistula in early oral feeding in laryngectomy patients. Am J Otolaryngol.

[8] Amin J, Ortlip TE, Cohen D (2020). The utility of barium swallow studies for evaluation of pharyngocutaneous fistula after total laryngectomy. Arch Otorhinolaryngol Head Neck Surg.

[9] Khoo MJ, Ooi AS (2021). Management of postreconstructive head and neck salivary fistulae: A review of current practices. J Plast Reconstr Aesthet Surg.

[10] Locatello LG, Licci G, Maggiore G, Gallo O (2021). Non-surgical strategies for assisting closure of pharyngocutaneous fistula after total laryngectomy: a systematic review of the literature. J Clin Med.

[11] Lee DJ, Lee YM, Park HJ (2021). Intraoperative botulinum toxin injection for superficial partial parotidectomy: a prospective pilot study. Clin Otolaryngol.

[12] McLean JN, Nicholas C, Duggal P (2012). Surgical management of pharyngocutaneous fistula after total laryngectomy. Ann Plast Surg.

[13] Sapundzhiev NR, Nikiforova LT, Spasova BH (2019). Endoscopic repair of pharyngocutaneous fistula following laryngectomy. Cureus.

[14] Vossoughinia H, Zarringhalam MA, Alamdari HD, Zinkanlou MA (2020). Fibrin glue in postlaryngectomy fistula-a case report. Iran J Otorhinolaryngol.

